# The Use of Reduced Models to Generate Irregular, Broad-Band Signals That Resemble Brain Rhythms

**DOI:** 10.3389/fncom.2022.889235

**Published:** 2022-06-13

**Authors:** Benjamin Ambrosio, Lai-Sang Young

**Affiliations:** ^1^Normandie Univ, UNIHAVRE, LMAH, FR-CNRS-3335, ISCN, Le Havre, France; ^2^The Hudson School of Mathematics, New York, NY, United States; ^3^Courant Institute of Mathematical Science and Center for Neural Science, New York University, New York, NY, United States; ^4^School of Mathematics, School of Natural Sciences, Institute for Advanced Study, Princeton, NJ, United States

**Keywords:** brain rhythms, gamma-band activity, E/I-conductances, slow-fast dynamics, randomly varying coefficients, power spectral densities

## Abstract

The brain produces rhythms in a variety of frequency bands. Some are likely by-products of neuronal processes; others are thought to be top-down. Produced entirely naturally, these rhythms have clearly recognizable beats, but they are very far from periodic in the sense of mathematics. The signals are broad-band, episodic, wandering in amplitude and frequency; the rhythm comes and goes, degrading and regenerating. Gamma rhythms, in particular, have been studied by many authors in computational neuroscience, using reduced models as well as networks of hundreds to thousands of integrate-and-fire neurons. All of these models captured successfully the oscillatory nature of gamma rhythms, but the irregular character of gamma in reduced models has not been investigated thoroughly. In this article, we tackle the mathematical question of whether signals with the properties of brain rhythms can be generated from low dimensional dynamical systems. We found that while adding white noise to single periodic cycles can to some degree simulate gamma dynamics, such models tend to be limited in their ability to capture the range of behaviors observed. Using an ODE with two variables inspired by the FitzHugh-Nagumo and Leslie-Gower models, with stochastically varying coefficients designed to control independently amplitude, frequency, and degree of degeneracy, we were able to replicate the qualitative characteristics of natural brain rhythms. To demonstrate model versatility, we simulate the power spectral densities of gamma rhythms in various brain states recorded in experiments.

## 1. Introduction

Rhythms, or oscillatory patterns of neural activity, occur ubiquitously in many parts of the central nervous system. One typically classifies them by their frequency bands. For example, β-band rhythms (12–30 Hz) are related to muscles and movements, and γ-rhythms (30–90 Hz) are implicated in information transfer and are associated with cognitive processes. Because brain rhythms have unique signatures and are relatively easy to record, they have been studied in hundreds of experimental article. The origins and functional roles of these rhythms, as well as their connections to various brain disorders, are active topics of current research, though much remains to be understood.

This article is not concerned with the biological origins of brain rhythms. Our interest lies in the signal itself, and our challenge is to generate mathematically signals that possess characteristics of rhythms produced naturally by the brain. We will focus on gamma rhythms, and for definiteness, we will base our study on gamma-band activity in the visual cortex, which has been the subject of detailed experimental studies e.g., Gray et al., 1989; Henrie and Shapley, 2005; Xing et al., 2012. Refer also to the review article by Cardin (2016), and the modeling article by Chariker et al. (2018).

Experimental data show that there are two aspects to the character of gamma rhythms: one is their oscillatory nature, and the other is their irregularity. In spite of their being called “a rhythm,” gamma rhythms are far from periodic in the sense of mathematics. There is a recognizable beat, to be sure, but spectral power density studies show that gamma rhythms are broad-band, with wandering frequencies and phases. Activity patterns are episodic; the beats are uneven in magnitude, degrading from time to time before the resumption of oscillatory behavior.

Several earlier theoretical studies (Ermentrout and Kopell, 1998; Brunel and Hakim, 1999; Brunel, 2000; Whittington et al., 2000; Tiesinga et al., 2001; Börgers and Kopell, 2003; Brunel and Wang, 2003; Fries, 2005) captured well the oscillatory behavior of gamma rhythms without delving into their irregular character. The broad-band, episodic nature of gamma rhythms was captured in biologically realistic models of the visual cortex, e.g., Rangan and Young (2013); Chariker and Young (2014); Chariker et al. (2018) using networks of hundreds to thousands of integrate-and-fire neurons. A more detailed exposition of earlier studies is given in the Section 6. In this article, we are interested in the following question: How can one generate, using reduced models or dynamical systems with few degrees of freedom, irregular rhythms with the variability in frequency and amplitude seen in natural brain rhythms?

We investigated the use of limit cycles perturbed by white noise as has been proposed by several authors and found that at the right noise level, these models do reproduce some gamma characteristics. The use of shear and anisotropic noise appeared to further improve the realism of the signal produced. We have found, however, that the most straightforward way to simulate the irregular character of gamma rhythms is to use a system of Ordinary Differential Equations (ODE) parameterized by quantities designed to control directly amplitude, frequency, and degree of degeneracy, and to allow these parameters to wander randomly.

For illustration, we present a specific example, consisting of a 2D slow-fast system inspired by the well-known FitzHugh-Nagumo and Leslie-Gower models. Viewing the two variables as Excitatory (E) and Inhibitory (I) conductances of typical neurons in a local population, this model produces results that resemble gamma-band activity in the real cortex. Bonuses of the model include moment-to-moment balancing of E and I-currents seen in experiments and known to theorists. To further demonstrate the versatility of this model, we challenged it to reproduce several sets of experimental data, including the power spectral densities and spectrograms recorded from awake vs. anesthetized monkeys, to simulate the changes in gamma-band activity associated with increased contrast and the repeated presentation of visual stimuli.

The rest of this article is organized as follows: In Section 2, we examine the use of limit cycles with noise to produce gamma-like rhythms. The main model is presented in Sections 3 and 4: Section 3 describes the deterministic model; a stochastic component is introduced in Section 4. In Section 5, we demonstrate the model's capabilities to simulate experimental data. This is followed by Section 6.

## 2. Rhythms Produced by Noisy Limit Cycles

As discussed in the Introduction, experimental data of gamma rhythms show a good deal of irregularity (Gray et al., 1989; Henrie and Shapley, 2005; Xing et al., 2012; Cardin, 2016); refer also to the modeling paper (Chariker et al., 2018). They show that gamma rhythms are far from periodic in the sense of mathematics but are broad-band, with wandering amplitudes and frequencies, their time courses punctuated by intermittent degradations in the rhythm. Reduced models that depict gamma rhythms as limit cycles (e.g., Ermentrout and Kopell, 1998; Fries, 2005) are not intended to possess irregular features. Several other authors (e.g., Brunel, 2000; Brunel and Wang, 2003) proposed to model gamma rhythms by limit cycles perturbed by white noise. Below we investigate the effectiveness of these stochastic models as a tool for simulating the irregular character of gamma rhythms, but first, we review quickly the idea of PSD, an important tool used by neuroscientists.

### 2.1. Quick Review of PSD

For periodic signals, Fourier coefficients provide the right mathematical tool for extracting the main frequencies. To capture the pseudo-periodicity of gamma rhythms, neuroscientists have used the following computational tool (Henrie and Shapley, 2005; Chariker et al., 2018).

The idea is to fix a time interval of suitable length *T* and to compute Fourier coefficients on [0, *T*] as if the signal was periodic with period *T*, i.e., let


(1) $$\begin{array}{l} \hat{f}\left(k\right)=\frac{1}{T}\int_{0}^{T}f\left(t\right)e^{-2i\pi k\frac{t}{T}}dt\;, \end{array}$$

and define the power concentrated at frequency *k* to be $|\hat{f}\left(k\right){|}^{2}$. To capture gamma-band frequencies, the time interval *T* is usually chosen to be between 100 and 500 ms: too short of an interval will fail to capture the relevant frequencies, and too long of an interval is ineffective since the signal is not truly periodic.

For a fixed initial time *t*_0_, we perform the computation above repeated with many time shifts, i.e., we sample the signal on [*t, t*+*T*] for *t* = *t*_0_, *t*_0_+*dt, t*_0_+2*dt*, ⋯  for small *dt*, and the squares of the computed Fourier coefficients, $|\hat{f}\left(k\right){|}^{2}$, are averaged over all of the samples to obtain what is called the *power spectral density* (PSD) and often referred as the Welch method in computational software, refer to Welch (1967).

### 2.2. Limit Cycles + White Noise

We consider here the system


(2) $$\begin{array}{l} \{\begin{array}{ccc} x^{\prime } & = & x-y-\left(x^{2}+y^{2}\right)x\\ y^{\prime } & = & x+y-\left(x^{2}+y^{2}\right)y \end{array} \end{array}$$

which in polar coordinates is


(3) $$\begin{array}{l} \{\begin{array}{ccc} r^{\prime } & = & r-r^{3}\\ \theta^{\prime } & = & 1 \end{array} \end{array}$$

In this system, the unit circle has period 2π, and it attracts all the trajectories distinct from the origin. To put the rhythm in the gamma range, we slow time by a factor of 3 to obtain a frequency of ~50 Hz. Adding white noise, we obtain the following system of stochastic differential equations (SDE):


(4) $$\begin{array}{l} \{\begin{array}{ccc} dx_{t} & = & x_{t}-y_{t}-\left(x_{t}^{2}+y_{t}^{2}\right)x_{t}+\mu_{x}dB_{t}^{1}\\ dy_{t} & = & x_{t}+y_{t}-\left(x_{t}^{2}+y_{t}^{2}\right)y_{t}+\mu_{y}dB_{t}^{2} \end{array} \end{array}$$

where (*B*^1^, *B*^2^) denotes a two dimensional Wiener process.

**Figure 1:**
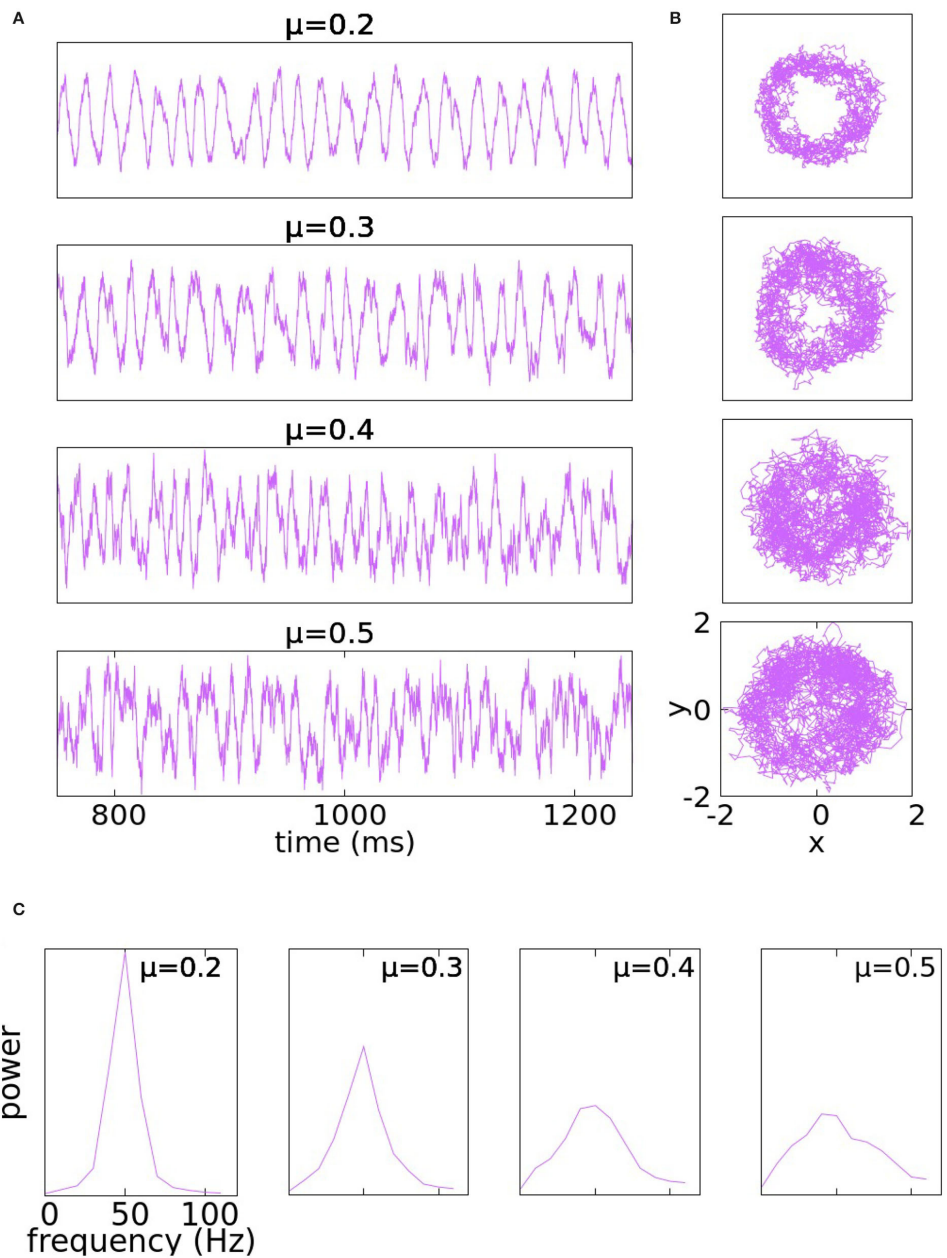
Panel **(A)** illustrates the traces of the *y*-coordinate for Equation (4) with μ_*x*_ = μ = μ_*y*_ at various levels of noise: from top to bottom the parameter μ takes the values 0.2, 0.3, 0.4, and 0.5. Panel **(B)** illustrates the trajectories of the same solutions in the *x*−*y* plane. At the bottom, in panel **(C)** are PSDs of the corresponding noise levels.

Typical solutions of Equation (4)- with μ_*x*_ = μ = μ_*y*_ at various levels of noise are shown in Figure 1. The traces are plots of the *y*-coordinate; the wriggly curves are sample paths of the SDE over many cycles. PSDs at corresponding noise levels are also shown.

At μ = 0.2, the peaks of the *y*-trace are somewhat irregularly spaced thanks to the noise, but the rhythm is too regular to resemble gamma rhythms produced by the real cortex. Increasing μ to 0.3 increases the amount of variability, but the rhythm is still too regular; in particular, it does not degenerate as can be seen by the empty spot near the origin in the phase plane trajectory. At μ = 0.4, the noise has significantly broadened the PSD (which is desirable), but local properties of Brownian paths also begin to manifest in the *y*-traces in the form of short rises and falls occurring at rapid successions. Such high-frequency oscillations on top of the main gamma rhythm are not typical of the behavior of membrane potentials in gamma activity. At μ = 0.5, the range of signal frequency becomes a little too broad, threatening to obstruct the main rhythm.

Our conclusion from the study above is that the addition of noise to purely periodic dynamics produces variability that goes some distance toward simulating the irregular character of gamma rhythms, but the use of a single parameter, namely μ= noise level, is too rigid: it is not irregular enough at low noise-levels and introduces undesirable features when noise-level is tuned too high.

### 2.3. Two Variants

Continuing to work with limit cycles subjected to white-noise forcing, we give two examples below to show the beneficial effects of an additional parameter.

Our first example adds *shear* to the deterministic dynamics. We introduce a new parameter α to Equation (4) to give


(5) $$\begin{array}{l} \{\begin{array}{ccc} dx_{t} & = & x_{t}-{\left(x^{2}+y^{2}\right)}^{\alpha }y_{t}-\left(x_{t}^{2}+y_{t}^{2}\right)x_{t}+\mu B_{1t}\\ dy_{t} & = & {\left(x^{2}+y^{2}\right)}^{\alpha }x_{t}+y_{t}-\left(x_{t}^{2}+y_{t}^{2}\right)y_{t}+\mu B_{2t} \end{array} \end{array}$$

The deterministic of this equation, reads in polar coordinates as,


(6) $$\begin{array}{l} \{\begin{array}{ccc} r^{\prime } & = & r-r^{3}\\ \theta^{\prime } & = & r^{2\alpha } \end{array} \end{array}$$

**Figure 2:**
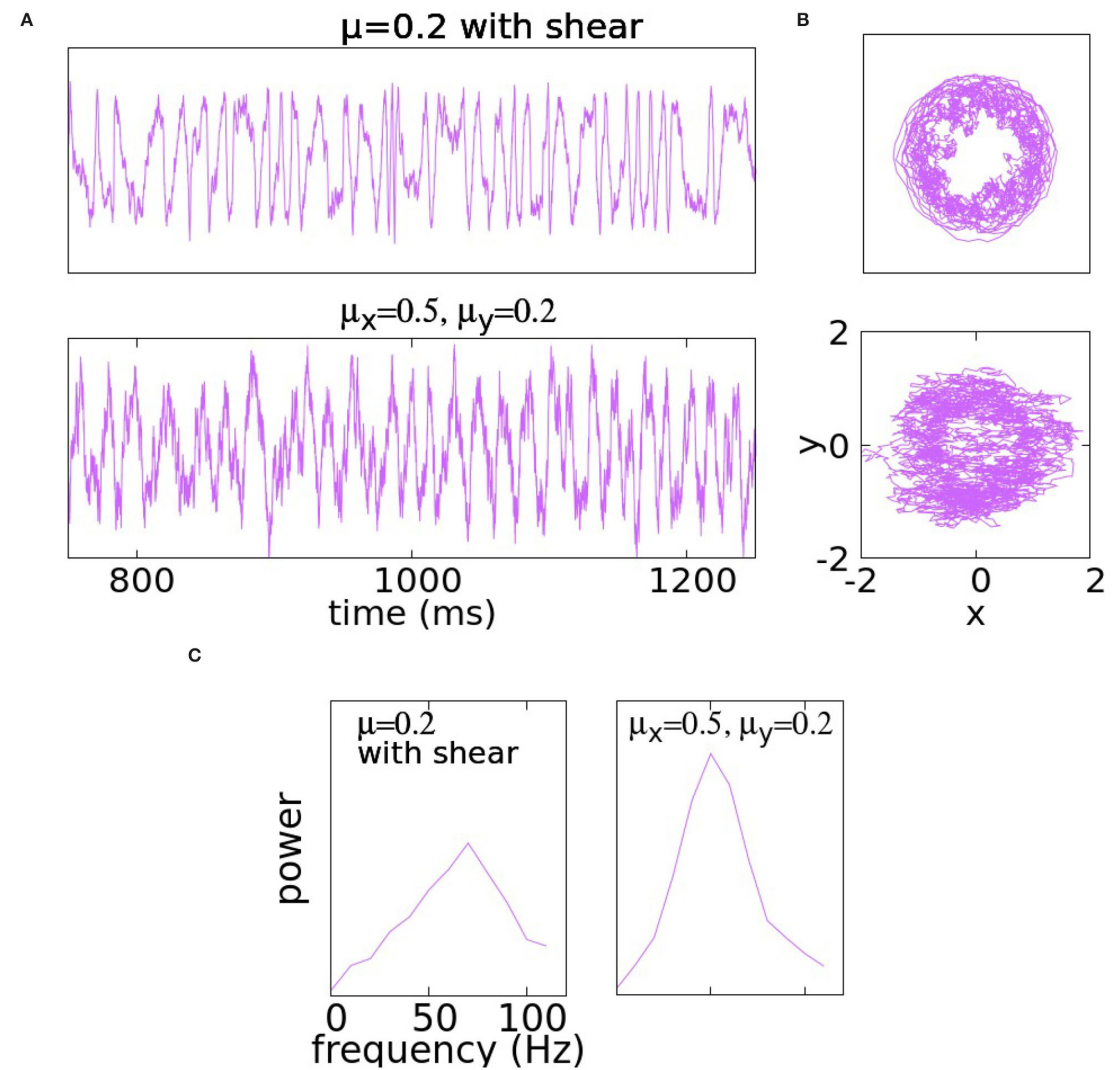
Panel **(A)** shows traces of the *y*-coordinate for Equation (5) (i.e., with shear) with μ_*x*_ = μ = μ_*y*_ = 0.2 in the first row and for (4) with asymmetric coefficients μ_*x*_ = 0.5 and μ_*y*_ = 0.2. Panel **(B)** illustrates the trajectories of the same solutions in the *x*−*y* plane. At the bottom, the PSD is illustrated in **(C)**.

The idea is that the farther a trajectory is from the center, the higher its angular velocity. Figure 2A shows the case of μ = 0.2 with α = 3. It is evident that at this noise level, shear produces significantly more varied behaviors, a fact confirmed by a much broader spectrum.

In a second example, we use *anisotropic noise*, i.e., the equation is as in (4) with μ_*x*_ = 0.5 and μ_*y*_ = 0.2. The idea is that the larger μ_*x*_ would add variability, while the smaller μ_*y*_ would not result in the unwanted Brownian structures in *y*-traces. These expectations are confirmed in Figure 2B.

** Remark 1**. *We conclude from the study above that because gamma signals are multi-faceted, to properly simulate them one needs to be able to control—independently—their frequencies, amplitudes, and degrees of degeneracy (i.e., the way the rhythm degrades from time to time and re-emerges). There has to be a mechanism for the system to switch from one regime to another at seemingly random times. Limit cycles with noise controlled by one or two parameters can reproduce certain aspects of the signal but do not possess sufficient flexibility. We propose in the sections to follow a system of ODEs with several parameters designed to control directly the properties we think are important and to produce the irregularity of gamma characteristics* *via* *stochastic motion in parameter space*.

## 3. Proposed Model: Deterministic Part

In this section and the next, we present our main model, consisting of a simple system of ODE with randomly varying coefficients. The deterministic part of the model, its key features, together with the quantities to be varied are presented in Section 3; the stochastic component is introduced in Section 4.

### 3.1. Model Equations and Basic Dynamical Features

**Figure 3:**
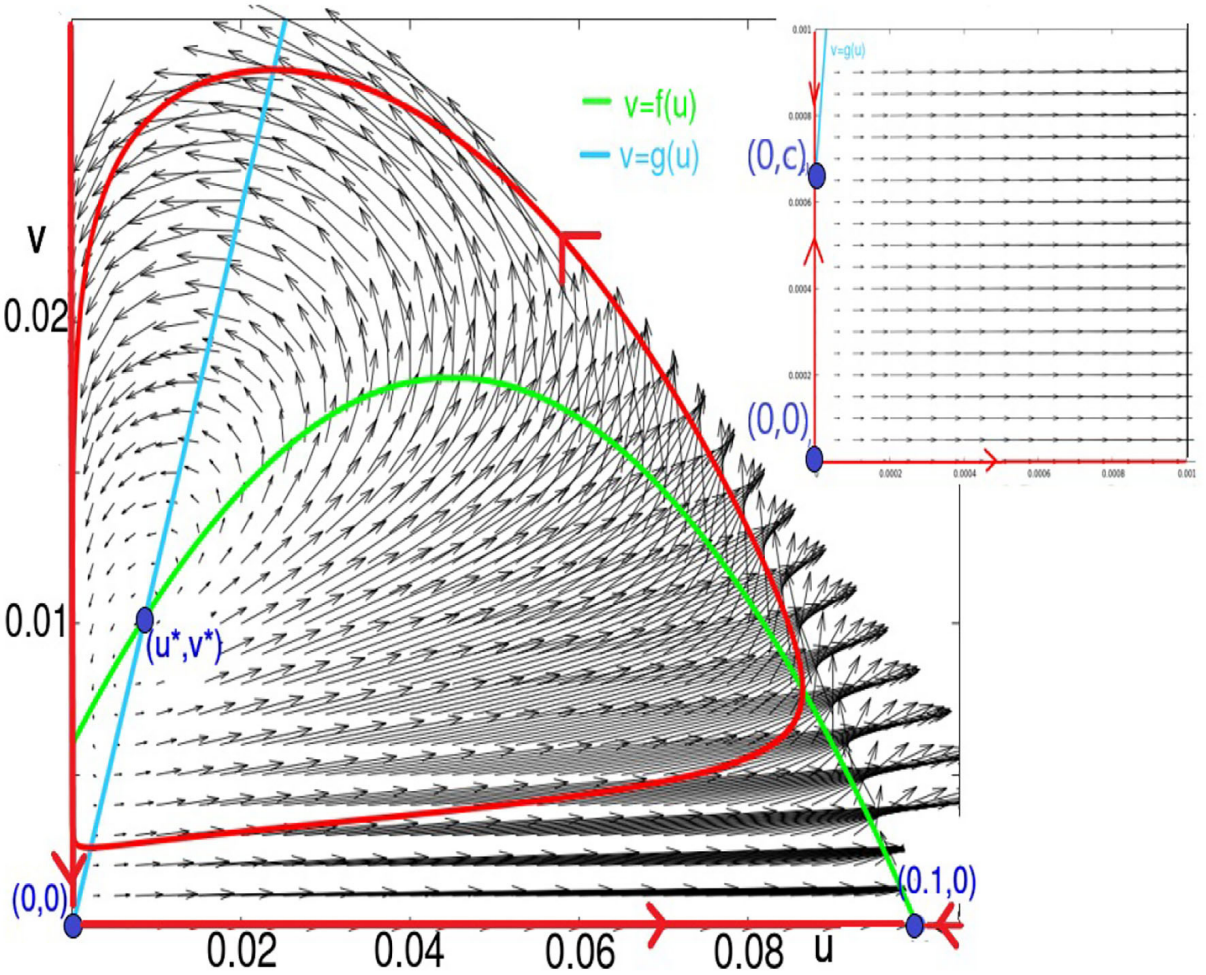
This figure shows the fixed points, nullclines, and vector field, as well as the trajectories lying in the sets *u* = 0, *v* = 0, and the limit-cycle for *K* = 60 and ϵ = 0.1. For these values, the parameter (*a*_2_, 0) is a saddle, and (u^*^, v^*^) is a source. The two fixed points (0, 0) and (0, *c*) are too close to be discernible in this figure. In the inset, we zoom in to visualize them: (0, 0) is a source, and (0, *c*) is a saddle.

**Figure 4:**
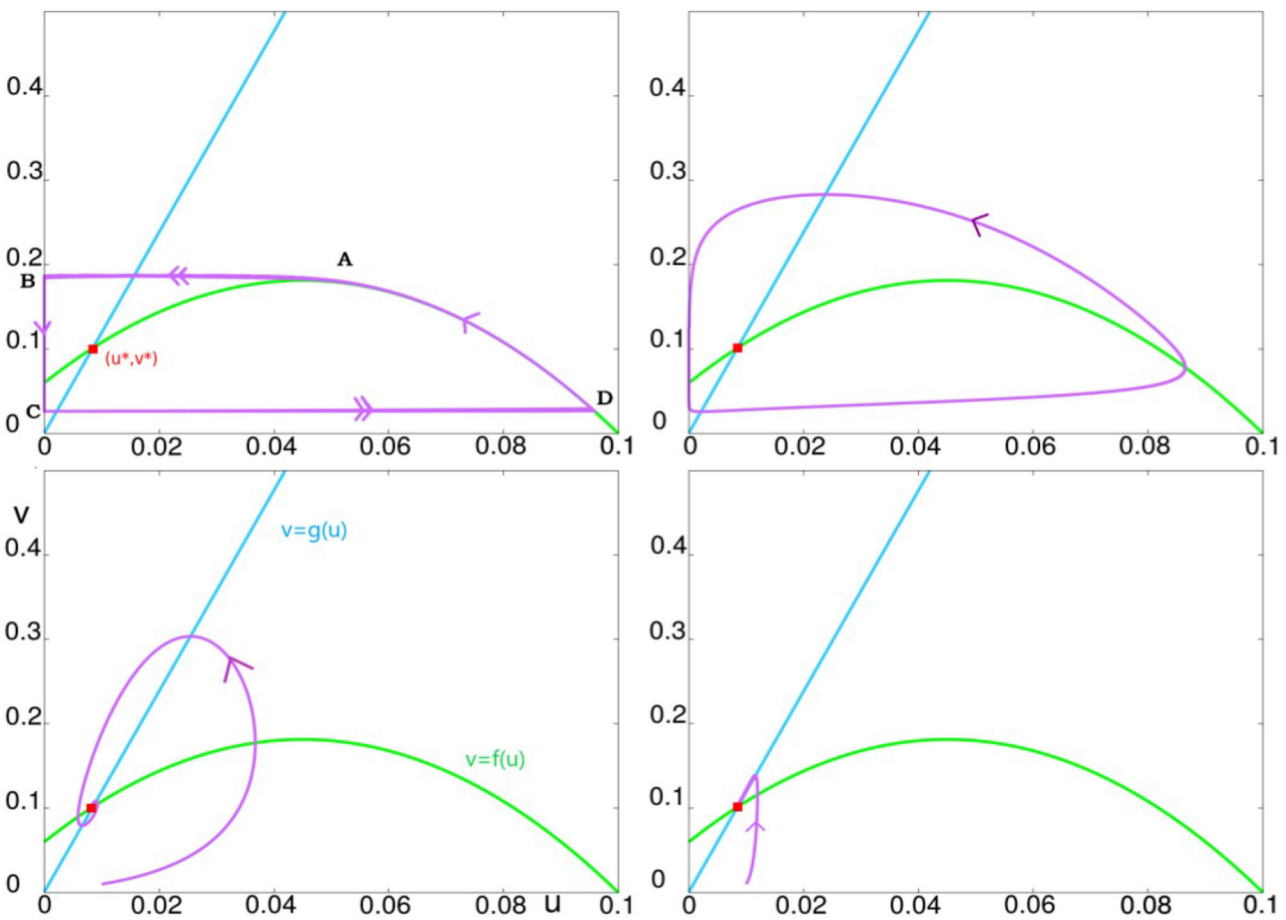
This figure illustrates the effect of varying ϵ, from small to large values. The parameter *K* is set to *K* = 60. The top left panel illustrates ϵ = 0.001, the top right, ϵ = 0.1, bottom left, ϵ = 1, and bottom right ϵ = 10.

Below *u* and *v* represent the absolute values of the E and I-conductances of a typical neuron in a local population. We propose that their dynamics be described by


(7) $$\begin{array}{l} \{\begin{array}{ccc} \epsilon u_{t} & = & u\left(-K\left(u-a_{1}\right)\left(u-a_{2}\right)-v\right)\\ v_{t} & = & \gamma v\left(bu-v+c\right) \end{array} \end{array}$$

where *a*_1_, *a*_2_, *b*, and *c* are fixed parameters with $a_{1}=-0.01,\;a_{2}=0.1,\;b=11.9,\;c=6.6\times 10^{-4}$. These values are chosen to reproduce the nullclines in Figures 3, 4, the dynamical significance of which are explained below. The key parameters are ϵ, γ, and *K*. They will be discussed at length below; for now, think of them as taking values in


ϵ∈[0.01,1],  γ∈[1,25]   and   K∈[30,100].

We will adopt the following notation:


$$\begin{array}{lll} F\left(u,v\right)=u\left(-K\left(u-a_{1}\right)\left(u-a_{2}\right)-v\right), & \; & G\left(u,v\right)=\gamma v\left(bu-v+c\right)\\ \;f\left(u\right)=-K\left(u-a_{1}\right)\left(u-a_{2}\right), & \; & g\left(u\right)=\left(bu+c\right)\;. \end{array}$$

The nullclines of Equation (7) are then given by


u=0,v=f(u)

for the first equation, and


v=0,v=g(u)

for the second equation. Note that the polynomial *f*(*u*) = −*K*(*u*−*a*_1_)(*u*−*a*_2_) reaches its maximum for *u* = 0.5(*a*_1_+*a*_2_) with a value of $0.25K{\left(a_{2}-a_{1}\right)}^{2}$.

Our choice of parameters allows only one intersection between the quadratic function *f*(*u*) and the linear function *g*(*u*) in the positive quadrant. This is ensured by the condition:


$$b\frac{a_{1}+a_{2}}{2}+c>0.25{\left(a_{2}-a_{1}\right)}^{2}K$$

which gives *K* < 176.8.

There are four stationary points in the positive quadrant, the region of interest. They are


$$\left(0,0\right),\left(0,c\right),\left(a_{2},0\right),\left(u^{*},v^{*}\right)$$

where u^*^ is the positive solution of


$$K\left(u-a_{1}\right)\left(u-a_{2}\right)+bu+c=0$$

and v^*^ = bu^*^+*c*. Refer to Figure 1, which gives a sense of the global dynamics and basic structures of the system.

Theorem 1 establishes rigorously the region of interest for this system.

** Theorem 1**. *The positive* (*u, v*)*-quadrant is invariant under the dynamics defined by Equation (7), and there exists a bounded absorbing set to which all solutions enter.*

*Proof*. The fact that the positive quadrant is positively invariant follows from the fact that *u* = 0, *v*_*t*_ = γ*v*(−*v*+*c*) and ϵ*u*_*t*_ = −*u*(*K*(*u*−*a*_1_)(*u*−*a*_2_)), *v* = 0, are solutions lying on the *u* and *v*-axes. For the existence of an absorbing set, we compute


(8) $$\begin{array}{l} \frac{d}{dt}\left(\epsilon u^{2}+v^{2}\right)=2[-Ku^{4}+K\left(a_{1}+a_{2}\right)u^{3}-Ka_{1}a_{2}u^{2}-u^{2}v\\ \;+b\gamma uv^{2}-\gamma v^{3}+c\gamma v^{2}] \end{array}$$

By using Young inequality, for *u, v* > 0,


$$uv\leq \frac{u^{p}}{p}+\frac{v^{q}}{q},\frac{1}{p}+\frac{1}{q}=1,$$

we can split the right hand side of (8) into a polynomial in *u* of degree 4, and a polynomial in *v* of degree 3, with both negative leading coefficients. Since the solution lies in the positive quadrant, by polynomial comparison, we obtain:


(9) $$\begin{array}{l} \frac{d}{dt}\left(\epsilon u^{2}+v^{2}\right)\leq -M\left(\epsilon u^{2}+v^{2}\right)+N \end{array}$$

where *M* > 0 and *N* > 0 can be chosen independently of initial conditions. Integrating (9) leads to the existence of an absorbing set attracting all trajectories.

** Remark 2**. *Equation (7) is inspired by the FitzHugh-Nagumo (FHN) and Leslie-Gower (LG) models. Starting from the FHN system, a well-studied system with a slow-fast structure, we replaced the cubic nullcline with a parabola in the first equation and added factors of* *u* *and* *v* *respectively in front of the first and second equations to constrain the solutions to the upper-right quadrant. Such modifications were necessary because in the classical FHN system, one variable describes voltage and the other is the so-called recovery variable, but the dynamics of voltage excursions and recovery follow different time courses than E- and I-conductances. Also, both variables in FHN take negative values, which the magnitudes of conductances do not. The variables in the LG system, on the other hand, have been shown (refer to Ambrosio et al., 2018) to have a dynamical character closer to that of the REI mechanism in Chariker et al. (2018). Relaxing the fast constraint (i.e., allowing ϵ to be larger) induces correlated dynamics in* *u* *and* *v* *that are strikingly similar to those observed in experimental and numerical simulations of conductance dynamics; compare, e.g., Figure 1 in Okun and Lampl (2009) and Figures 2B,E in Chariker et al. (2018).*

### 3.2. Varying the Parameter ϵ

Recall that we have three parameters: ϵ, γ, and *K*. We first explain the role of ϵ, fixing for now γ = 1, and studying the dynamics as ϵ is varied for each value of *K*. The dynamics of the system from ϵ very small to very large for *K* = 60 (a fairly typical value of *K*) are summarized in Figure 2. For ϵ ≪ 1, Equation (7) describes a slow-fast system with a limit cycle as can be seen in the top two panels of Figure 2. As ϵ is increased, this limit cycle turns into a sink somewhere between ϵ = 0.1 and ϵ = 1. For large ϵ, e.g., at ϵ = 10, one can show that the critical attractive manifold is the line Δ:*v* = *g*(*u*). All the trajectories reach this line fast and then follow it slowly toward the fixed point (u^*^, v^*^) as can be seen in the bottom right panel.

Below we will give the analysis for ϵ very small, as well as the Hopf bifurcation that takes the limit cycle to the sink.

#### 3.2.1. The Case of ϵ≪1

For ϵ small enough a slow-fast analysis allows to compute the limit-cycle up to an *O*(ϵ) order. The behavior can be described geometrically as follows. We denote by C the curve *v* = *f*(*u*). For ϵ small enough, a trajectory starting from the right side of C (i.e., at any point on the curve between *A* and *D*), will increase along the curve (*v*_*t*_ > 0 there) until it reaches the maximum point $A=\left(\frac{a_{1}+a_{2}}{2},f\left(\frac{a_{1}+a_{2}}{2}\right)\right)$. Refer to Figure 2 top left panel. This is a jump point, refer to Krupa and Szmolyan (2001), i.e., from there the trajectory leaves C and goes at high speed to reach a neighborhood of the point $B=\left(0,f\left(\frac{a_{1}+a_{2}}{2}\right)\right)$. After that, since at first *u*_*t*_ < 0, the trajectory remains stuck near the line *u* = 0. It goes down (*v*_*t*_ < 0) until it crosses the point (0, *f*(0)) = (0, −*Ka*_1_*a*_2_), at which point *u*_*t*_ becomes positive. This is a fold point but not a jump point, refer to Krupa and Szmolyan (2001). Dynamics near this point have been analyzed in Ambrosio et al. (2018). Refer also to Wang and Zhang (2019) and references therein cited. The trajectory continues to follow the axis *u* = 0 until it reaches a point *C* on the axis *u* = 0 which is significantly below (0, *f*(0)). Here, there is the possibility of the so-called *canard* phenomenon, refer to Benoît et al. (1981); Krupa and Szmolyan (2001); Szmolyan and Wechselberger (2001). At *C*, the trajectory leaves the axis *u* = 0 and goes very quickly toward the point *D* on C with the same ordinate as *C*. This gives a qualitative description of the limit-cycle. For ϵ sufficiently small, precise statements can be rigorously deduced from Geometrical Singular Perturbation Theory. Good reviews can be found in Hek (2010); Jones (1995); Kaper (1999); Krupa and Szmolyan (2001).

Let Γ′ be the closed curve defined by:


$$\Gamma^{\prime }=\left[A,B\right]\cup \left[B,C\right]\cup \left[C,D\right]\cup \zeta$$

where ζ⊂C is the arc from *D* to *A*.

** Theorem 2**. *For ϵ > 0 sufficiently small, there is a limit cycle* Γ *within distance* *O(ϵ) of* Γ′.

For proof of the uniqueness of the limit-cycle in the case ϵ small, refer to Wang and Zhang (2019).

We point out that the system defined by (7) provides a simple example, in a Neuroscience context, in which canard solutions emerge and can be computed explicitly. As a result of the polynomial expression of the vector field, the computations performed in Ambrosio et al. (2018) become simpler and explicit around the point (0, *f*(0)).

#### 3.2.2. Hopf Bifurcations

As indicated in Section 2.1, the range of ϵ of interest is [0.1, 1], and it is in this range of ϵ that the limit cycle turns into a sink as shown in Figure 2. We now give more detail on this bifurcation, specifically, the Hopf bifurcation that occurs at (u^*^, v^*^) where (u^*^, v^*^) is the unique fixed point in the interior of the positive quadrant; refer to Section 2.1.

The Jacobian matrix at fixed points is


$$J=\left(\begin{array}{cc} \frac{1}{\epsilon }\left(-3Ku^{2}+2K\left(a_{1}+a_{2}\right)u-Ka_{1}a_{2}-v\right) & -\frac{1}{\epsilon }u\\ bv & -2v+\left(bu+c\right)\; \end{array}\right).$$

Substituting in v^*^ = bu^*^+*c*, we obtain at (u^*^, v^*^), that


$$J_{\left(u^{*},v^{*}\right)}=J^{*}=\left(\begin{array}{cc} -\frac{1}{\epsilon }Ku^{*}\left(2u^{*}-\left(a_{1}+a_{2}\right)\right) & -\frac{1}{\epsilon }u^{*}\\ b\left(bu^{*}+c\right) & -\left(bu^{*}+c\right) \end{array}\right),$$

which gives


$$\det\left(J^{*}\right)=\frac{1}{\epsilon }u^{*}\left(bu^{*}+c\right)\left(K\left(2u^{*}-\left(a_{1}+a_{2}\right)\right)+b\right)$$

while


$$tr\left(J^{*}\right)=-\frac{1}{\epsilon }Ku^{*}\left(2u^{*}-\left(a_{1}+a_{2}\right)\right)-\left(bu^{*}+c\right).$$

From the above expressions, we deduce the following proposition.

** Proposition 1**. *For* *K* ∈ [30, 100], *det*(J^*^) > 0. *It follows that for each* *K* *there exists a value of ϵ at which a Hopf bifurcation occurs. This value is given by:*


$$\epsilon =Ku^{*}\frac{a_{1}+a_{2}-2u^{*}}{bu^{*}+c}.$$

We close this section with an application of the Poincare-Bendixon theorem to our system.

** Theorem 3**. *Each trajectory starting in the region* {*u* > 0, *v* > 0} *either converges to* (u^*^, v^*^) *or evolves toward a limit-cycle. For* $\epsilon <Ku^{*}\frac{a_{1}+a_{2}-2u^{*}}{bu^{*}+c}$, *it converges toward a limit-cycle.*

*Proof*. The proof follows from the analysis of the nullclines and the nature of fixed points.

### 3.3. Dependence of Dynamics on the Parameters ϵ, *K*, and γ

Continuing to keep γ = 1, we first examine the dynamics of Equation (7) as functions of *K* and ϵ. Simulation results are shown in Figure 4. Notice first that these results are consistent with those in Figure 3 with regard to increasing ϵ for fixed *K*. What is new here is the effect of varying *K* for each ϵ. Figure 4 shows clearly that larger *K* corresponds to larger excursions by *u* and *v*. This means

(i) When solutions are attracted to a limit cycle, the limit cycle has a larger diameter for larger *K*; and
(ii) Whether solutions eventually tend to a limit cycle or a sink, this attracting set is located closer to *u* = 0, *v* = 0 for smaller values of *K*.

Finally, we examine the effect of varying γ. From the equations, it is clear that trajectories of Equation (7) will trace out the same curves as long as γϵ remains constant; and that varying γ keeping γϵ fixed corresponds to changing the speed with which one moves along these curves. For example, at *K* = 60, for values of ϵ = 0.1 and γ = 1, numerical simulation gives a limit cycle with period ~44 ms (equivalently frequency around 22 Hz). For ϵ = 0.01, γ = 10, the period becomes 4.4 ms (frequency around 225 Hz).

** Proposition 2**. *For each fixed* *K*, *the curves traced out by the trajectories of Equation (7) depend only on ϵγ. Fixing* *K* *and ϵγ, and varying γ, velocities are proportional to γ; in particular, the frequency of the limit cycle is proportional to γ*^−1^.

The meaning and main general effects of the variation of parameters ϵ, *K*, and γ in Equation (7) can be summarized as follows:

- *Increasing* ϵ *changes the dynamical regime from one with a limit cycle in a slow-fast system to one with an attractive fixed point;*
- *K* *controls the sizes of the excursion of* (*u, v*) *in the system's oscillatory behavior: in general, the larger* *K, the larger the excursions; while*
- *For each fixed value of* ϵγ*, the magnitude of* γ *controls the frequencies of the limit cycle*.

As we will show momentarily, these are the parameters we need to vary to produce the irregularities seen in gamma rhythms.

## 4. Proposed Model: Stochastic Components

As discussed in the Section 1, there are two facets to gamma rhythms as observed in the real cortex: one is their oscillatory nature; the other is their irregular, episodic character. The deterministic system in Section 2 provided the underlying oscillations. Here, we create irregularity by adding randomness to the deterministic model. Instead of adding white noise to the system of ODE, we have found that allowing its key parameters (as described in Section 3) to drift freely, performing random walks within designated parameter ranges, produces better results. This wandering parameters paradigm is especially effective for modeling dynamical behavior that samples different regimes, drifting from one regime to another after seemingly random time durations. By choosing a deterministic model capable of supporting the relevant dynamical regimes at different parameter values, one can control the sampling of different regimes by controlling the way the model's parameters wander.

As discussed earlier, gamma-band activity is quite varied in character: when the rhythm is robust, the dynamics appear to be following periodic orbits, the amplitudes and frequencies of which vary with time in a way that is partially history-dependent. When the rhythm degrades, it is as though the trajectory is near a (weakly) stable equilibrium. Our deterministic model supports these regimes; moreover, we have learned in Section 3.3 how to switch between sinks and limit cycles, and how to vary the amplitudes and frequencies of the cycles by changing parameters. By allowing these parameters to wander, we ensure that the amplitudes and frequencies of the gamma cycles will wander. We then choose the ranges of parameters and the speeds with which they vary as we see fit.

**Figure 5:**
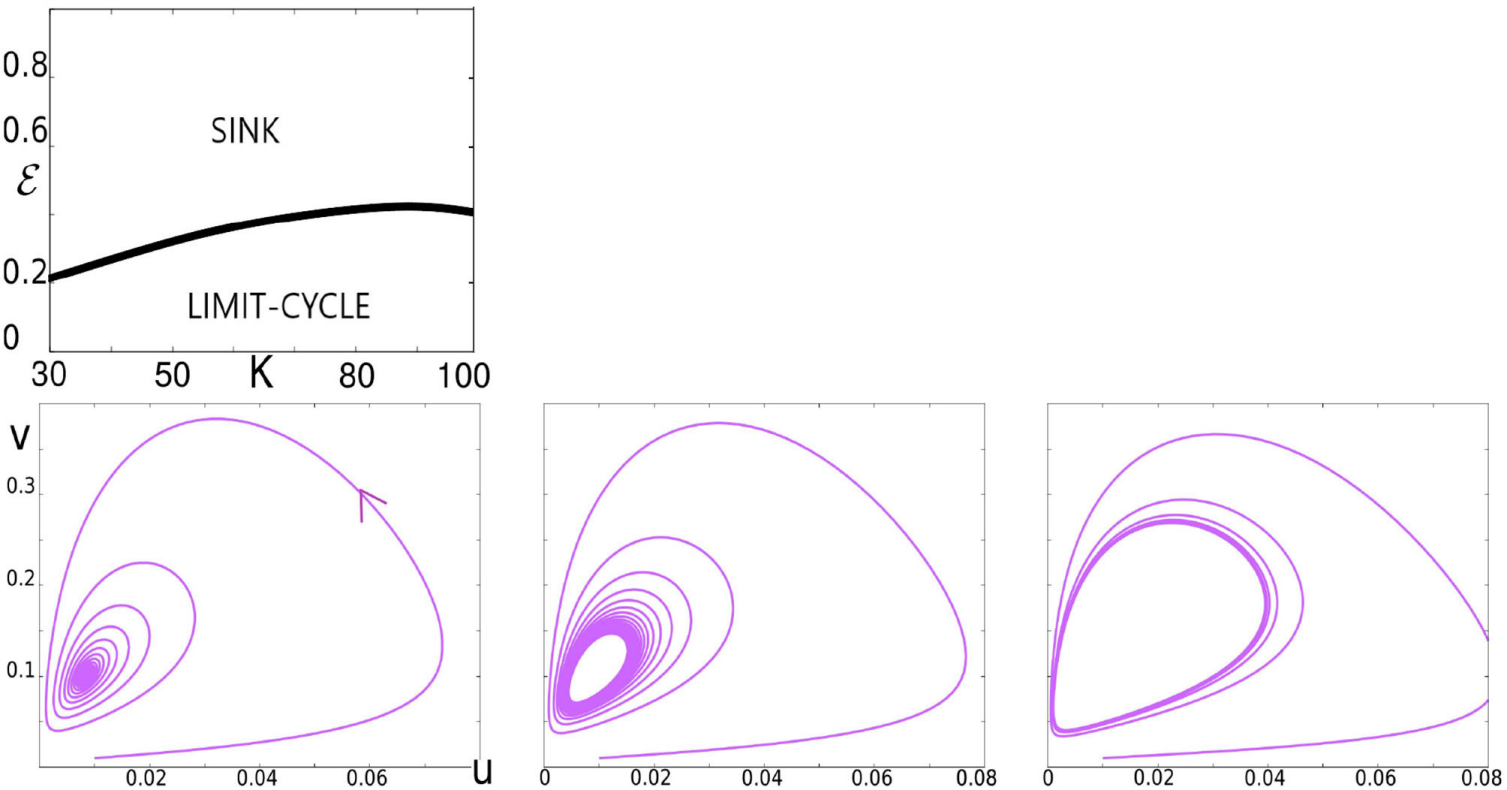
Illustration of the Hopf bifurcation. In the top panel, we have plotted the Hopf bifurcation diagram in the *K*, ϵ plane. The bottom panels illustrate the bifurcation for *K* = 60 as ϵ decreases. Left: ϵ = 0.4, a trajectory spiraling toward a sink. Middle: ϵ = 0.36, trajectories accumulating on a limit cycle following the sink's loss of stability. Right: ϵ = 0.3, the limit cycle growing in size.

In more detail, we first specify parameter ranges [*K*_min_, *K*_max_], [ϵ_min_, ϵ_max_], and [*f*_min_, *f*_max_] for *K*, ϵ, and ϵγ, respectively. For example, for the simulations shown in Figure 5, we used [*K*_min_, *K*_max_] = [30, 100], [ϵ_min_, ϵ_max_] = [0.04, 0.1], and [*f*_min_, *f*_max_] = [0.2, 0.5]. Let $U_{i}^{1},U_{i}^{2},U_{i}^{3},i=1,2,\cdots \;,$ be independent random variables uniformly distributed on [−1, 1]. Starting from initial values of *K*, ϵ, and γ within the specified ranges, we update these parameters every 0.1 ms. At the *i*th step, we let


$$K=K\left(1+0.1U_{i}^{1}\right),$$

constraining *K* to [*K*_min_, *K*_max_] according to the rule that if $U_{i}^{1}=u$ and *K*(1+0.1*u*) falls outside of [*K*_min_, *K*_max_], then we set *K* = *K*(1−0.1*u*). Next, we update ϵ by letting


$$\epsilon =\epsilon +0.01U_{i}^{2}\;$$

constraining ϵ to [ϵ_min_, ϵ_max_] as before.

Finally, we set


$$\gamma =\gamma +0.1U_{i}^{3}$$

if ϵγ ∈ [*f*_min_, *f*_max_]. If not, if ϵγ > *f*_max_ we set


$$\gamma =\frac{f_{\max}}{\epsilon }-0.1\times 0.5\left(1+U_{i}^{3}\right)$$

and if ϵγ < *f*_min_, we set


$$\gamma =\frac{f_{\min}}{\epsilon }+0.1\times 0.5\left(1+U_{i}^{3}\right)$$

**Figure 6:**
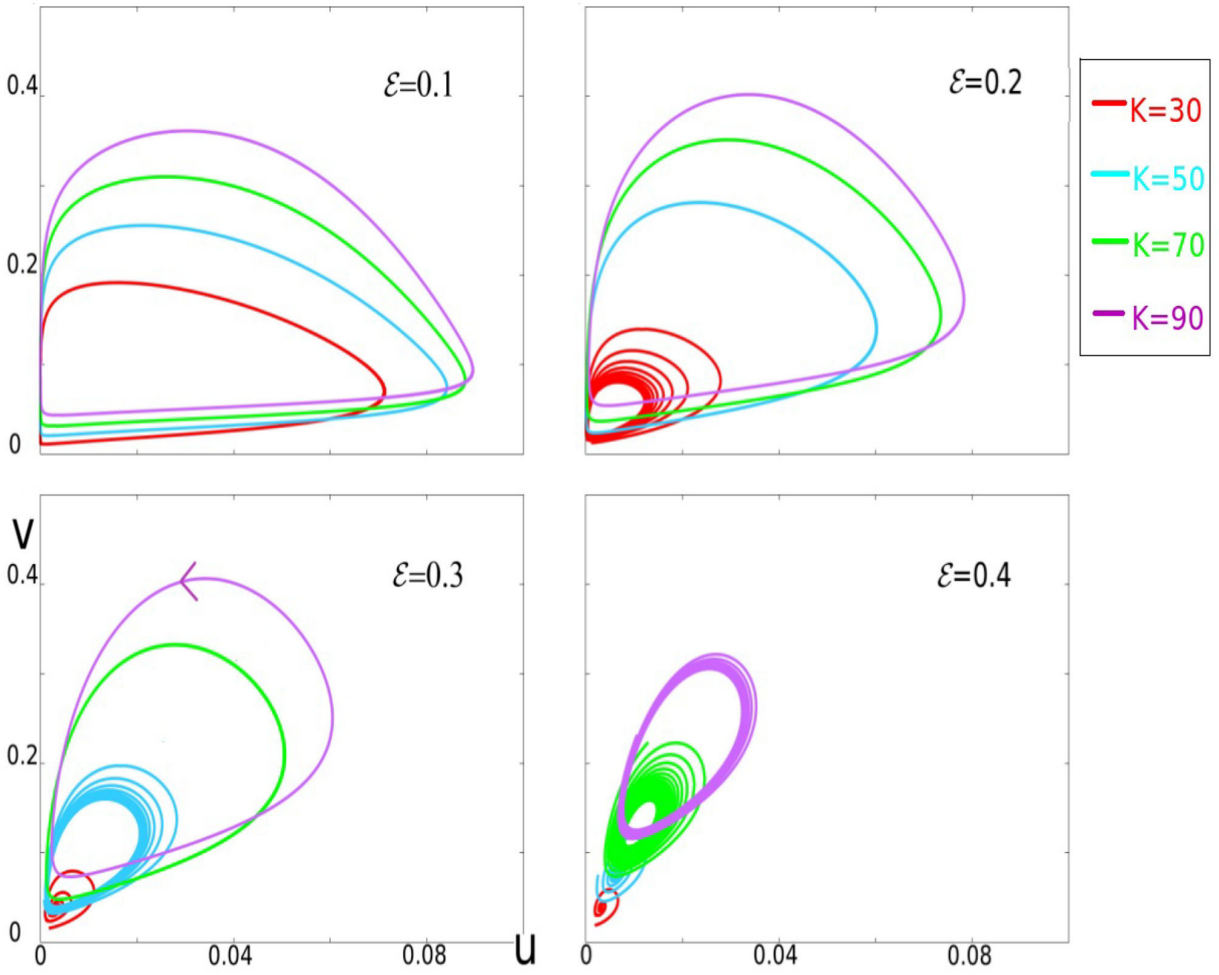
This figure gives a panorama of sample trajectories within the parameter's range of interest. Four panels corresponding to ϵ = 0.1, 0.2, 0.3, and 0.4 are shown. In each panel, trajectories for different values of *K* are depicted in different colors: *K* = 30 (red), 50 (cyan), 70 (green), and 90 (purple). For each value of ϵ, the larger *K*, the larger excursions in the phase-space.

Recall that it is the product ϵγ that determines the curves traced out by the trajectories of the system (Proposition 2), and ϵγ ∈ [0.2, 0.5] corresponds to ϵ ∈ [0.2, 0.5] in Figures 3–6, where γ was set = 1. Thus, to simulate gamma rhythms, the parameters above are chosen so that most but not all of the time, the dynamics are oscillatory. Once parameters that produce suitable qualitative behaviors are located, it is generally simpler to adjust the values of *u, v*, or the mean frequencies of the oscillations by modifying slightly the two equations of Equation (7) (e.g., by inserting a scaling coefficient in front).

**Figure 7:**
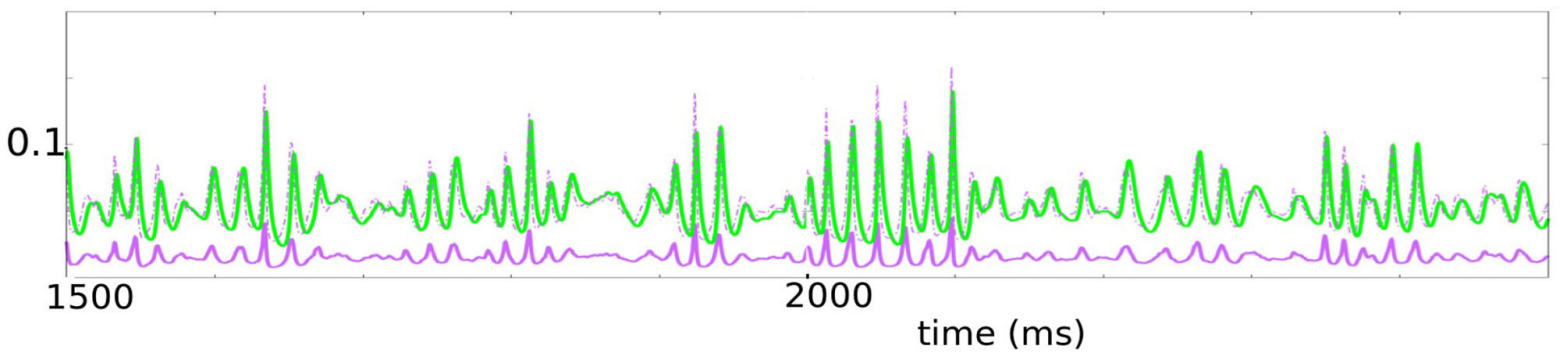
This figure represents the evolution of the stochastic version of the system (7). The parameters used are *K* ∈ [30, 50], ϵ ∈ [0.04, 0.1], γϵ ∈ [0.2, 0.5]. We plotted ū = 1.96*u*+0.00672 in solid purple, to be thought of as representing E-conductance, *v* in green, representing I-conductance. Since the ratio of E-current to E-conductance is roughly three to four times that of the ratio of I-current to I-conductance, we have plotted also 3.5ū in dashed purple. Note the tight relationship between 3.5ū and *v*.

Figure 7 shows a solution to the stochastic version of Equation (7). The irregular nature of the rhythm is clearly visible, and it possesses features remarkably similar to those in experimental data, refer to e.g., Burns et al. (2011) (Figure 1B): One sees the trajectory switching between robustly periodic and more degenerate regimes as the parameters controlling degeneracy are varied; the use of random walks with adjustable speeds has allowed us to control the degree of history dependence. The amplitudes and frequencies sampled are also controllable.

As discussed earlier, a bonus of this model is that it is more than just a phenomenological model of gamma: the two variables *u* and *v* suitably adjusted simulate the E and I-conductances of a typical neuron in a local population under drive. By definition, the E-current entering a neuron is defined to be its E-conductance times a factor proportional to the distance of membrane potential to the E-reversal potential, and the same is true for I-currents. As this factor for E-current is 3 to 4 times that for I, we have also plotted (in dash) a graph that is 3.5 times the height of the E-conductance. Modulo a multiplicative constant, then, the dashed purple and green plots can be thought of as approximations of E and I-currents, respectively, and it is striking how the two currents track one another.

We remark on the tightness with which the green plots (I-current) follow the dashed purple plots (E-current). There is a well-known theory of balanced states (van Vreeswijk and Sompolinsky, 1998) that asserts that in the limit as system size tends to infinity, E-currents and I-currents are balanced when averaged over time. Experimental results of Okun and Lampl (2008) and subsequent theory (Denève and Machens, 2016) and modeling paper (Chariker et al., 2018) show that much more than that is true, namely that these currents are in fact roughly balanced from moment to moment, not just when averaged over time. The tight relationship between our dashed purple and green curves in Figure 5 captured well this phenomenon.

## 5. Demonstration of Model Versatility

Below we give three examples to demonstrate that the ODE system with stochastically varying coefficients presented in Sections 3 and 4 can be used to generate signals that simulate rhythmic activity in the real cortex.

### 5.1. Example 1. Gamma Rhythms for Awake vs. Anaesthetized Monkey

The first set of experimental results we used to challenge our model was that reported in Xing et al. (2012). In this article, the authors studied gamma rhythms in awake vs. anesthetized monkeys. LFP from V1 (the primary visual cortex) in response to high contrast sinusoidal grating patches were recorded and the resulting data was analyzed. After the initial power increase (which we do not model), peak gamma frequency was found to be about of 60 Hz in the awake and 40 Hz in the anesthetized monkeys studied (Figure 1 of Xing et al., 2012). Time frequency analysis of single trials confirmed that oscillations in the awake animal were faster (Figure 2). Also, for anesthetized monkeys, the signal was weaker, having an amplitude about 60% that of the awake (Figure 1). In addition to the PSD, which describes spectral power averaged over time, the authors of Xing et al. (2012) (Figure 2) studied temporal structures of gamma-band activity. They found intermittent bursts of activity lasting for small fractions of a second suggesting some short term history dependence.

**Figure 8:**
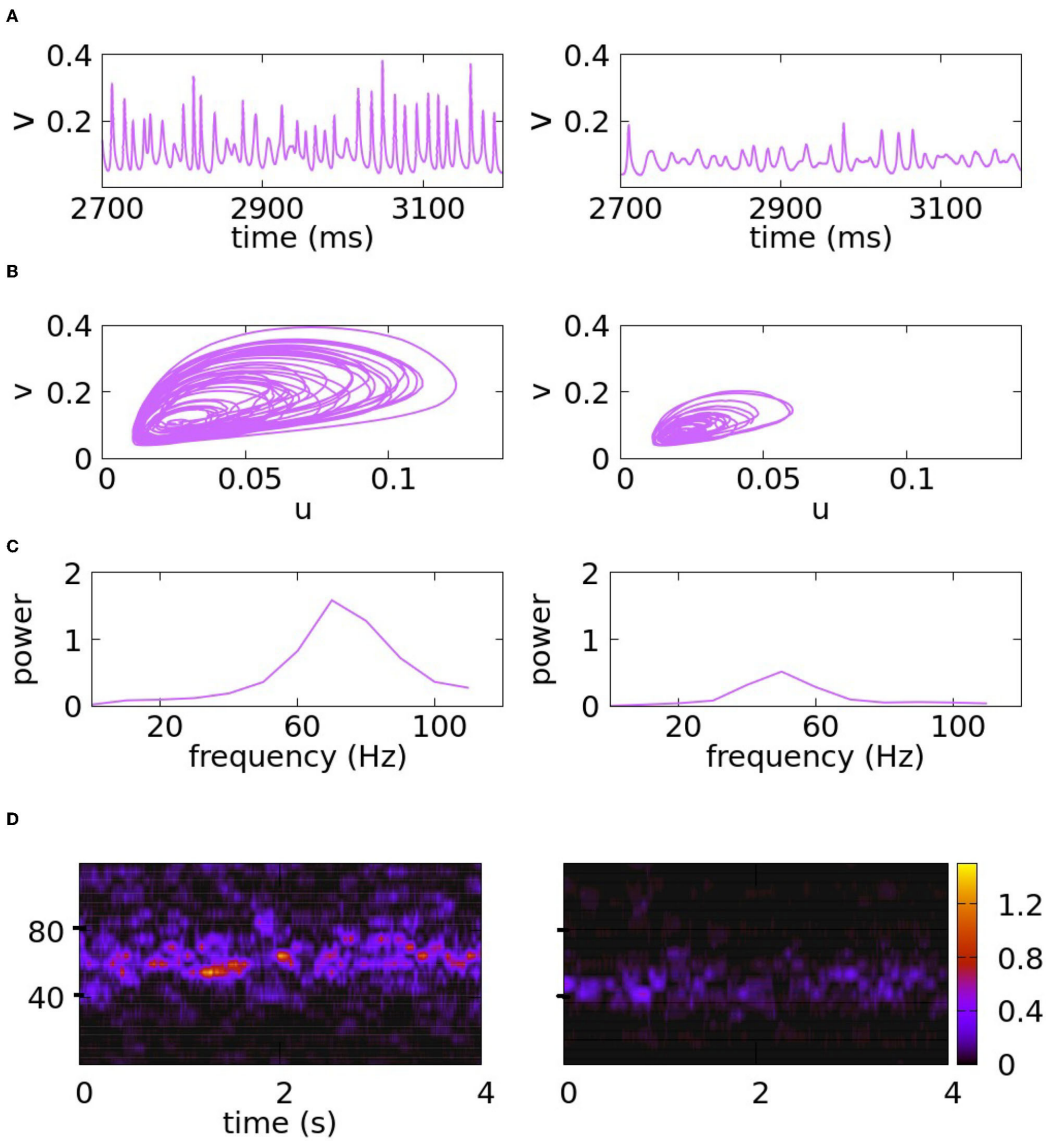
Reproducing signals with the characteristics of awake and anesthetized monkeys. The figures on the left side correspond to the awake state, and the figures on the right side correspond to the anesthetized state. The figures result from the simulation of Equation (7). In **(A)**, the traces of *v* are plotted as a function of time. In **(B)**, we represent the trajectories in the phase space. Panel **(C)** corresponds to the PSD and panel **(D)** to the spectrograms. The parameters are as follows: *K*_*m*_ = 50, *K*_*M*_ = 90, ϵ_*m*_ = 0.07, ϵ_*M*_ = 0.16, *f*_*m*_ = 0.35, *f*_*M*_ = 0.4 for the left side. For the right side, the following changes are made: *K*_*m*_ = 40, *K*_*M*_ = 68, ϵ_*m*_ = 0.08, ϵ_*M*_ = 0.18.

As proof of concept, we challenged our model to produce two signals with the characteristics of brain rhythms of awake and anesthetized monkeys as reported in the experimental article above. Our goal is not a perfect match with data but to demonstrate how these quantities can be manipulated through parameter selection in a model like the one presented in Sections 3 and 4. Time traces, PSDs, and spectrograms (Fourier power computed on shifted time intervals and plotted as a function of time, refer to Section 2.1) for two signals intended to simulate these two very different cortical states are presented in Figure 8; the exact parameters used are given in the legend. The amplitudes and peak frequencies of the PSD plots are in agreement with the data and the spectrogram shows small bursts of activity.

### 5.2. Example 2. PSD in Primate Visual Response: High vs. Low Contrast

**Figure 9:**
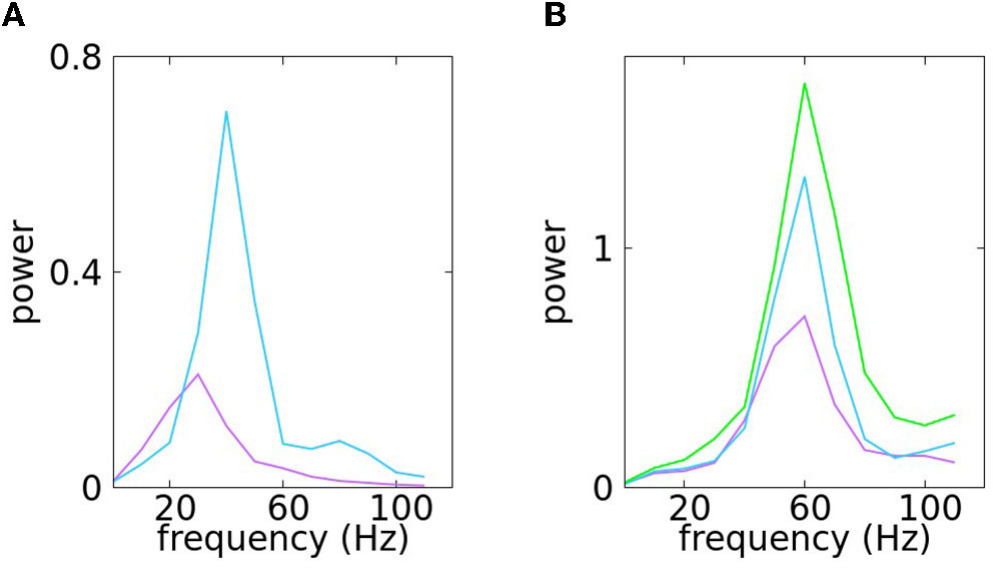
In **(A)**, we reproduce low contrast vs. high contrast as in Jia et al. (2013). This results for simulation of equation (7) with: *K*_*m*_ = 25, *K*_*M*_ = 55.0, ϵ_*m*_ = 0.09, ϵ_*M*_ = 0.19, ϵγ ∈ [0.35, 0.4] for low contrast and *K*_*m*_ = 40, *K*_*M*_ = 70.0, ϵ_*m*_ = 0.11, ϵ_*M*_ = 0.21, ϵγ ∈ [0.35, 0.4] for high contrast. In **(B)**, we illustrate the increase of power related to repeated stimulations as in Brunet et al. (2014). The set of parameters is as follows, low power: *K*_*m*_ = 40, *K*_*M*_ = 75.0, ϵ_*m*_ = 0.075, ϵ_*M*_ = 0.155, ϵγ ∈ [0.35, 0.4], mean power: *K*_*m*_ = 45, *K*_*M*_ = 80.0, ϵ_*m*_ = 0.09, ϵ_*M*_ = 0.16, ϵγ ∈ [0.35, 0.4], and *K*_*m*_ = 50, *K*_*M*_ = 90.0, ϵ_*m*_ = 0.09, ϵ_*M*_ = 0.19, ϵγ ∈ [0.35, 0.4] for high power.

It is well-known to visual neuroscientists that in primate contrast response, peak frequency and spectral power increase with contrast (Henrie and Shapley, 2005). For definiteness, we challenged the model to reproduce characteristics of the signals in Jia et al. (2013). Figure 2C of Jia et al. (2013) shows that in response to a large grating (10° in diameter), peak frequency increased from about 30 to 44 Hz, and stimulus-induced gamma power was enhanced more than 3-fold as the contrast was increased from about 6 to 50%. Selecting suitable parameters from our model, we were able to build two signals having these spectral characteristics. They are shown in Figure 9A.

### 5.3. Example 3. Effect of Stimulus Repetition on Gamma-Band Activity

It is shown by Brunet et al. (2014) that the repeated presentation of a visual grating stimulus to monkeys resulted in a steady increase of visually induced gamma-band activity in V1 and V4 (and in the synchronization of the two rhythms in these two areas). The authors proposed this as a plausible way to maintain effective stimulus signaling in the face of dwindling firing rates, presumably due to adaptation. Figure 1 in Brunet et al. (2014), which shows LFP traces and power spectra from a recording session with an awake monkey, shows peak frequencies increasing slightly but hovering mostly around 60 Hz, consistent with the values in Example 1. A striking feature here is that the PSDs become increasingly sharply peaked, with significant increases in gamma power at these frequencies with stimulus repetition. In Figure 9B, we present the PSD of 3 signals with these properties generated by our model.

The examples above demonstrate that the two dimensional ODE system with stochastically varying coefficients presented in Sections 3 and 4 of this article is sufficiently flexible that through parameter selection, one can reproduce, on demand, a variety of characteristics observed in gamma-band rhythms in the real cortex.

## 6. Discussion

Oscillatory behaviors are among the most widely observed dynamical phenomena in physiology. They occur in the spontaneous beating of heart cells (Glass et al., 1984), in central pattern generators in animal locomotion (Cohen et al., 1988), and in calcium oscillations that underlie a plethora of cellular responses (Thul et al., 2008). For more examples refer to Glass and Mackey (1988); Françoise (2005); Winfree (2000). Physiological processes can also be more complex than just purely oscillatory, sometimes they can even be mildly chaotic, as has been observed in several studies, such as Bondarenko (1994); Freeman (1987); van Vreeswijk and Sompolinsky (1998), and Lin et al. (2012). Brain rhythms, gamma-band oscillations, in particular, are neither purely oscillatory nor chaotic but somewhere in between, and their simulation has been much studied by theorists. We review below a sample of the main results prior to this study.

### 6.1. Previous Study on Models of Gamma Rhythms

An early and well-known model is PING (Whittington et al., 2000; Börgers and Kopell, 2003); similar models include (Ermentrout and Kopell, 1998; Tiesinga et al., 2001) among others. These models were the first to use non-linear dynamics to explain gamma rhythms. The original PING model produces highly regular population spikes, capturing successfully the oscillatory behavior of gamma rhythms but not their irregular character. There were several follow-up studies e.g., (Borgers et al., 2005; Börgers, 2017) in which network models were used to produce more nuanced spike patterns.

Another body of study that received much attention is (Brunel and Hakim, 1999; Brunel, 2000). In these studies, the authors started with networks of sparsely coupled integrate-and-fire neurons, and let system size tend to infinity while keeping the number of connections an infinitely small fraction of system size. Arguing that in such a limit distinct neurons are likely to have disjoint sets of presynaptic cells, the authors of Brunel and Hakim (1999); Brunel (2000) modeled neuronal dynamics by an equation consisting of a deterministic part describing meanfield activity plus a Gaussian noise that is independent of the neuron to neuron, and gamma rhythms were modeled as regimes following a supercritical Hopf bifurcation. These are the first reduced models of gamma rhythms that we know of. Another much cited article is by Brunel and Wang (2003). Here, the authors assumed that gamma rhythms consisted of purely periodic motion plus a noise term, and focused on the dependence of the period on various factors.

Experimental studies from the last 15 years brought to light some intriguing features of gamma rhythms produced by the real cortex, stressing their broad-band nature (Henrie and Shapley, 2005). They show that the amplitudes of the oscillations in local field potentials can be quite large (Henrie and Shapley, 2005), far from regimes that emerge following Hopf bifurcations. Another feature of interest revealed by experimental results is that the power and frequencies of the oscillations *wander* (Xing et al., 2012), with the same patterns often persisting for tens, sometimes up to 200 ms, indicative of some form of short-term memory.

More recently, a number of detailed biological network models have appeared showing that gamma rhythms with the properties above occur naturally as a consequence of Excitatory-Inhibitory interaction in local neuronal processes, calling attention to concepts such as *multiple firing events* (MFE) (Rangan and Young, 2013) and *recurrent excitation-inhibition* (REI) (Chariker et al., 2018). The analysis in these recent articles provided an understanding, at least qualitatively, of how both the oscillatory and irregular characters of gamma rhythms come about.

### 6.2. What This Article Is About: Goals and Conclusion

This article is concerned not with physiological processes associated with gamma rhythms but with the mathematical properties of the signal itself. As these rhythms are naturally produced and have very intriguing signatures, we sought to understand how they can be produced using reduced neuronal models defined by low dimensional dynamical systems. As noted above, reduced models of gamma rhythms have been studied before, but how well they capture the broad-band, irregular nature of gamma rhythms had not been evaluated up until now.

Thus, we began in Section 2 with a study of the main group of reduced models in the literature, namely those described by periodic dynamics perturbed by white noise (Brunel, 2000; Brunel and Wang, 2003). We found that these models do reproduce some aspects of the irregular side of gamma behavior but the multi-faceted nature of these behaviors required more multi-dimensional control. To that end, we proposed, in Sections 3 and 4, a model inspired by the FitzHugh-Nagumo system. We do not claim that the model we proposed is the only viable model, far from it, but the following properties of this model are of note: As parameters are varied, the dynamical regimes described range from stable equilibria to Hopf bifurcations to robust limit cycles. Other parameters offer direct control of the amplitudes and frequencies of the periodic regimes. We found also that varying parameter randomly but continuously, such as random walks in parameter space, capture more realistically the wandering nature of gamma characteristics.

In the final section, to demonstrate versatility we challenged the model to reproduce several sets of experimental data, and the model performed satisfactorily. We remark that the modeling approach employed here can be used to study other rhythms provided that we adjust augment the model to accommodate the characteristics of the rhythm in question. A challenging example is signals with superposition of rhythms. In this case, an idea would be to consider two coupled models of ours, the strength of coupling depending on the interaction of these rhythms.

## 7. Methods

Simulations of the system (7), in the deterministic case, were performed using a standard *RK*4 method with a time step of *dt* = 0.01, on the time interval [0, *T*], with *T* = 5, 000*ms*. The code is a personal code written by the first author in the *C*++ language.

More explicitly, this means that, classically, the time interval [0, *T*] is divided into sub-intervals [*jdt*, (*j*+1)*dt*], *j* ∈ {0, ..., *T*/*dt*−1}, and the solution of (7) is approximated by the sequence (*u*_*j*_, *v*_*j*_)_*j* ∈ {0, ..., *T*/*dt*}_ with (*u*_*j*_, *v*_*j*_) computed from (*u*_*j*−1_, *v*_*j*−1_) by using the *RK*4 iteration.

For the stochastic version of Equation (7), the numerical method remains the same, with the specification that for *j* = 10, 20, ⋯  the parameters *K*, ϵ, and γ are changed randomly according to the description given in paragraph 3, *i*.*e*., let $U_{i}^{1},U_{i}^{2},U_{i}^{3},i\in \left\{1,2,....,49900\right\}$ be independent random variables uniformly distributed on [−1, 1]. For *j* = 10*i, i* ∈ {1, ..., 49990}, we let


$$K=K\left(1+0.1U_{i}^{1}\right),$$

constraining *K* to [*K*_min_, *K*_max_] according to the rule that if $U_{i}^{1}=u$ and *K*(1+0.1*u*) falls outside of [*K*_min_, *K*_max_], then we set *K* = *K*(1−0.1*u*). Next, we update ϵ by letting


$$\epsilon =\epsilon +0.01U_{i}^{2}\;$$

constraining ϵ to [ϵ_min_, ϵ_max_] as before. Finally, we set


$$\gamma =\gamma +0.1U_{i}^{3}$$

if ϵ* γ* ∈ [*f*_min_, *f*_max_]. If not, if ϵ* γ* >*f*_max_ we set


$$\gamma =\frac{f_{\max}}{\epsilon }-0.1\times 0.5\left(1+U_{i}^{3}\right)$$

and if ϵγ < *f*_min_ we set


$$\gamma =\frac{f_{\min}}{\epsilon }+0.1\times 0.5\left(1+U_{i}^{3}\right).$$

## Data Availability Statement

The original contributions presented in the study are included in the article/supplementary material, further inquiries can be directed to the corresponding author.

## Author Contributions

BA and L-SY set up the mathematical model, performed the research, provided the mathematical analysis, and wrote the manuscript. BA did the numerical simulations. All authors contributed to the article and approved the submitted version.

## Funding

Part of the research has been funded by Région Normandie France, ERDF (European Regional Development Fund) XTERM, CNRS International Emerging Actions program, and the Hudson School of Mathematics. Part of the research of L-SY was funded by NSF Grants 1901009 and 1734854.

## Conflict of Interest

The authors declare that the research was conducted in the absence of any commercial or financial relationships that could be construed as a potential conflict of interest.

## Publisher's Note

All claims expressed in this article are solely those of the authors and do not necessarily represent those of their affiliated organizations, or those of the publisher, the editors and the reviewers. Any product that may be evaluated in this article, or claim that may be made by its manufacturer, is not guaranteed or endorsed by the publisher.

## Abbreviations

E: Excitatory
I: Inhibitory
LIF: leaky integrate and fire
PING model: Pyramidal-interneuronal network gamma model
ODE: Ordinary Differential Equation
FHN: FitzHugh-Nagumo
REI: Recurrent Excitation Inhibition
SDE: Stochastic Differential equation
PSD: Power Spectral Density
KAM: Kolmogorov-Arnold-Moser.
